# Exploring the role of white matter connectivity in cortex maturation

**DOI:** 10.1371/journal.pone.0177466

**Published:** 2017-05-17

**Authors:** Cecilia L. Friedrichs-Maeder, Alessandra Griffa, Juliane Schneider, Petra Susan Hüppi, Anita Truttmann, Patric Hagmann

**Affiliations:** 1 Department of Radiology, Centre Hospitalier Universitaire Vaudoise (CHUV), Lausanne, Switzerland; 2 Signal Processing Laboratory (LTSS), Ecole Polytechnique Fédérale de Lausanne (EPFL), Lausanne, Switzerland; 3 Clinic of Neonatology and Follow-up, Department of Pediatrics, Centre Hospitalier Universitaire Vaudois (CHUV), Lausanne, Switzerland; 4 Division of Neurology, The Hospital for Sick Children, University of Toronto, Toronto, Canada; 5 Division of Development and Growth, Department of Pediatrics, University of Geneva, Geneva, Switzerland; Institute of Psychology, Chinese Academy of Sciences, CHINA

## Abstract

The maturation of the cortical gray matter (GM) and white matter (WM) are described as sequential processes following multiple, but distinct rules. However, neither the mechanisms driving brain maturation processes, nor the relationship between GM and WM maturation are well understood. Here we use connectomics and two MRI measures reflecting maturation related changes in cerebral microstructure, namely the Apparent Diffusion Coefficient (ADC) and the T1 relaxation time (T1), to study brain development. We report that the advancement of GM and WM maturation are inter-related and depend on the underlying brain connectivity architecture. Particularly, GM regions and their incident WM connections show corresponding maturation levels, which is also observed for GM regions connected through a WM tract. Based on these observations, we propose a simple computational model supporting a key role for the connectome in propagating maturation signals sequentially from external stimuli, through primary sensory structures to higher order functional cortices.

## Introduction

Although most of its cellular components are formed by mid-gestation, the human brain continues to mature into postnatal life [1,2]. This reflects the development of the organized structural connectivity network, which is at the basis of neural processing [3], for a review). These maturation processes consist of myelination of both white (WM) and gray matter (GM), but also of dendritic arborization, growth of terminal axon arborization and synaptic pruning in the cortex. Adequate completion of this developmental phase is crucial as insults during this period result in impaired neurodevelopmental outcome [4].

Distinct sequential patterns of GM and WM maturation are described. Cortical GM maturation is related to the development of brain functions. Evidence shows that primary sensory and motor areas differentiate [5,6] and reach mature dendritic structure [7,8], adequate intra-cortical myelination [9,10] and mature macroscopic architecture [11] before higher-order processing areas such as fronto-parietal cortices. WM axonal maturation mainly follows a spatial gradient, with myelination beginning in central and caudal areas and progressing towards polar and rostral locations [12–15].

Evidence from histology and in vivo imaging studies suggests that a relationship exists between the maturation of axonal WM connections and local GM structural features (e.g., emergence of gyri and sulci) [16–18]. Moreover, the WM maturation level of distinct functional circuits was related to corresponding functional scores, such as performance in working memory [19] and language-related tasks [20]. Nonetheless, so far, the large-scale mechanisms driving the brain maturation patterns, and the relationship between the development of GM and WM structures, remain unclear.

While early patterns of neural cell differentiation and axonal guidance are mainly driven by genetic molecular cues [21], refinement and maturation of the produced coarse neural circuits including synaptic, axonal and dendritic outgrowth and pruning also depend on neural activity [22]. This early electrical activity is spontaneous during early development and progressively becomes experience-driven during the postnatal period [23]. Starting from the last trimester of pregnancy [24], it acts in synergy with molecular cues [25] and is fundamentally important as it provides trophic support for neuronal survival and endogenous guidance for activity dependent wiring [26–28]. Recent studies outlined a key role of action potentials for promoting myelination [29,30]. Animal studies provide evidence that early feed-forward sensory experience plays a key role in shaping local GM structure [31,32] and excitatory/inhibitory network architecture [33–35]. Altogether, these findings suggest that WM connections relay essential signals involved in cerebral plasticity and development. Accordingly, the propagation of nervous signals from one cortical area to the next through WM tracts could promote maturation of both the GM and the outgoing WM connections, and ultimately contribute to define the overall spatio-temporal patterns of brain maturation.

From these considerations, we make a simplifying hypothesis that: (i) WM axonal pathways play a key role in shaping maturation of cortical structures, and (ii) maturation propagates in a feed-forward fashion originating from primary cortical sensory areas, whose activations are initially spontaneous and later on driven by external sensory inputs, and spreading through the WM network toward higher-order processing areas over time.

Recently developed MRI sequences sensitive to microstructural changes in the cerebral cortex and the WM (diffusion tensor, T1-mapping) have emerged as sensitive assays to study developmental related changes in human brain [36–40]. Our study is designed in two parts. First, we use two of these measures, namely the Apparent Diffusion Coefficient (ADC) and the T1 relaxation time (T1). We then extensively compare these parameters within GM regions and within their incident WM axonal tracts. In the second part, we propose a computational model of brain maturation based on a random walk process, which reproduces empirical measures of cortical and WM maturation, and supports the hypothesis that the WM connectivity network plays a key role in conveying biological signals essential for brain maturation.

## Materials and methods

### Subjects

Neonatal scans from 9 very prematurely born infants (4 males, mean gestational age: 27.3 weeks ± 1.0 day) acquired at term equivalent age (TEA, mean age at scan 41.0 weeks ± 2.0 days) were selected from a prospective cohort of neonates born before 30 weeks of gestation conducted between February 2011 and May 2013 in the level III neonatology unit at the University Hospital of Lausanne, Switzerland [40]. This cohort included solely children considered as ‘cerebral low-risk’, meaning that they showed no evidence for severe intraventricular haemorrhage grade III-IV and/or parenchymal haemorrhagic infarction, no high grade periventricular leucomalacia, no congenital malformations or genetic abnormalities and had normal neurological assessment at TEA according to the Hammersmith Neonatal Neurologic Examination from [41]. The local ethical committee of the Canton de Vaud approved the current study and parental written informed consent was obtained. Inclusion of the subjects in our study was based on (i) data quality, (ii) no substantial brain lesions on TEA MRI and (iii) absence of significant cognitive and psychomotor delays at 18 months of corrected age. Single subjects’ characteristics are reported in S1 Table.

### MRI data acquisition

Structural and diffusion-weighted MR images were obtained with a 3T scanner (MAGNETOM TrioTim, Siemens Healthcare, Germany) and a dedicated 8-channel baby head coil (NOMAG, LMT Lammers) integrated in an MR-compatible incubator. Structural scans were acquired using a double-echo MP2RAGE sequence with the following parameters: flip angle 4 degrees, TI1/TI2/TR = 900/2200/4000 ms, TE = 3.17 ms, GRAPPA R = 2, TA = 4.58 min. Two image volumes per echo time were obtained (at the 1st and 2nd inversion time INV1b and INV2b) and two morphological high resolution T1-weighted images were reconstructed: a bias-free T1-weighted image (*flatImg*) and a T1 relaxation map (*T1map*) [42]. The acquisition was sagittal with a FOV of 190 mm (feet-head) x 179 mm (ant-post) and a matrix of 256 x 241 voxels yielding an in-plane resolution of 0.7 x 0.7 mm^2^ and 96 slices of 1.2 mm.

The diffusion-weighted acquisitions were performed using Diffusion Tensor Imaging (DTI) with a twice-refocused spin echo EPI sequence with TR/TE = 5200/84 ms and a spatial resolution of 8 mm^3^ (2x2x2 mm^3^) isotropic with an in-plane matrix of 96x96 voxels and 43 slices. Five b0-images and 30 diffusion-weighted images with b-value of 1000 s/mm^2^ were acquired with varying directions. Acquisition time was about 3 minutes.

### Anatomical brain tissue segmentation and parcellation

MP2RAGE *flatImg* scans were segmented into brain tissue classes (GM including cortical and subcortical structures, WM and cerebrospinal fluid) based on single voxel signal intensity and probabilistic tissue location priors from the publicly available neonate brain atlas by Shi et al. [43] using *SPM* software [44]. The obtained probabilistic maps were thresholded empirically to obtain binary tissue masks. The GM volume was subdivided into 90 anatomical ROIs, i.e. 45 per hemisphere, according to the Shi atlas. Each ROI was dilated in order to reach the GM-WM border, and possible holes and discontinuities in the ROI masks were corrected using mathematical morphology operations. An expert neuroradiologist reviewed the intermediary mask images at each processing step. S1 Fig shows the resulting anatomical brain masks for the 9 subjects. The MP2RAGE *flatImg* volumes were linearly registered to the first B0 volume in order to bring the brain tissue masks into the diffusion space.

### Diffusion MRI processing and brain connectivity estimation

Diffusion-weighted MRI volumes were registered to the first B0 scan (rigid-body transformation, 6 degree of freedom) to correct for head motion. Next, these images were corrected for eddy current distortions using the FSL eddy correction software [45]. Tensor based reconstruction of diffusion information was performed using the *dtirecon* tool from Diffusion Toolkit [46], with the gradient table updated for the rigid-body registration performed earlier. Whole brain tractography was performed using the deterministic streamline algorithm implemented in the *Connectome Mapping Toolkit* [47,48] and using standard parameters. 32 streamlines were initiated in each WM voxel and propagated along the two opposite directions given by the local tensor orientation. Streamlines propagation was stopped if one of the following criteria was met: (i) touching the WM-GM boundary, or (ii) angle between diffusion directions in neighbouring voxels larger than 60 degrees. Fibers shorter than 6 mm and fibers not reaching the WM-GM border were discarded. Note that due to variability in head size, no stopping criterion was used for the maximal fiber length. Each reconstructed fiber trajectory reaching the WM-GM interface represented an anatomical connection between the corresponding pair of GM regions. Finally, structural connectivity matrices representing subject-wise brain networks were constructed by counting the number of streamlines connecting each pair of cortical and subcortical regions. For all analyses, we used a group-representative connectivity matrix computed by considering only the connections present for at least 50% of the subjects (excluding intra-ROI connections) and averaging the corresponding connection weights. This arbitrary group threshold was chosen based on previous work showing that a threshold between 50 and 60% yielded the best approximation of the number of existing connections and an acceptable balance between elimination of false positives and prevention of false negatives [49].

### WM and GM maturation parameters

In this study, we used two MRI measures reflecting maturation related changes in cerebral microstructure, namely the Apparent Diffusion Coefficient (ADC) and the T1 relaxation time (T1). ADC corresponds to the mean diffusivity, which reflects the level of diffusion restriction. In the brain ADC is a biomarker of maturation as it decreases with age [50,51] and reflects changes in axonal diameters and packing as well as myelination [52]. T1 relaxation changes with the concentration of macromolecules and lipids and therefore is also a marker of brain maturation and myelination [40,53,54]. The MP2RAGE sequence enables rapid, robust and high resolution of maps of T1 relaxation as described in [42,55]. ADC maps were computed from reconstructed DTI data using Diffusion Toolkit *dtirecon* function. Next, average ADC and T1 values were computed for each GM ROI, and for each WM connection between pairs of GM regions by averaging the voxel-wise scalars along the connecting streamline trajectories. Mean ADC and T1 values were calculated over (i) voxels belonging to each GM ROI, and (ii) WM connections incident to this ROI. In order to account for partial volume effects, GM voxels whose value differed by more than one standard deviation from the ROI mean were discarded from the analysis. From the 4005 possible ROI pairs, we restricted our analysis to the group wise connectivity matrix described in the previous section yielding a total of 450 connected and 3555 unconnected ROI pairs. Pearson correlation analyses were used to assess the relationship between ADC and T1 values in ROIs and over their incident WM connections. ROIs were sorted into four different groups according to the function-related GM maturation sequence described in the literature: group *SUB* included the subcortical structures (Basal Ganglia, the Thalami, the Amygdala and the Hippocampus), Group *PRIM* comprised primary sensory and motor cortices, Group *SEC* secondary processing areas and Group *TER* higher order tertiary areas (S2 Table).

### Maturation random walk model

A computational random walk model [56] was used to simulate the evolution of the brain tissue’s maturation as a particle walking along the structural brain pathways. Starting in one of the primary sensory cortex regions (*rolandic operculum*, *calcarine cortex*, *postcentral gyrus*, *paracentral lobule*, *heschl gyrus*), a particle travels from one ROI (network vertex) to another along anatomical connections (edges) of the brain network. The weight *w*_*ij*_ of an edge connecting two vertices *i*, *j* of the brain network is equal to the normalized number of streamlines connecting the two vertices (i.e., the number of streamlines connecting vertices *i* and *j* divided by the overall number of connecting streamlines resulting from the tractography algorithm). In our computational model, the probability of moving from a vertex *i* at time point *t* to a vertex *j* at time point *t+1* is proportional to the weight *w*_*ij*_ of the edge *(i*,*j)*. Furthermore, particles are not allowed to travel back to the previous region (no-back walk). Formally, if the walk is at vertex *i* at time *t*, the probability of taking a step along one of the edges attached to *i* is pij= wij∑k∈Nitwik, with $N_{i}^{t}$ set of neighbours of vertex *i*, excluding the vertex situated at the previous step *t-1*. We considered the number of particle transits through each GM region or WM connection, at each step of the random walk, as a score of local maturation. Random walks were initiated in each one of the ten primary cortex, and propagated for ten random-walk steps (RWSs). This process was repeated 1000 times for each seeding area, and mean GM and WM maturation scores were computed. Finally, spatiotemporal maturation patterns yielded by each seeding area were summed, illustrating whole brain maturation scores. For each RWS, the relationship between experimental ADC and T1 values on the one side, and total brain maturation scores on the other side, was assessed with a Spearman’s correlation analysis.

## Results

### Structural connectivity properties

**Fig 1:**
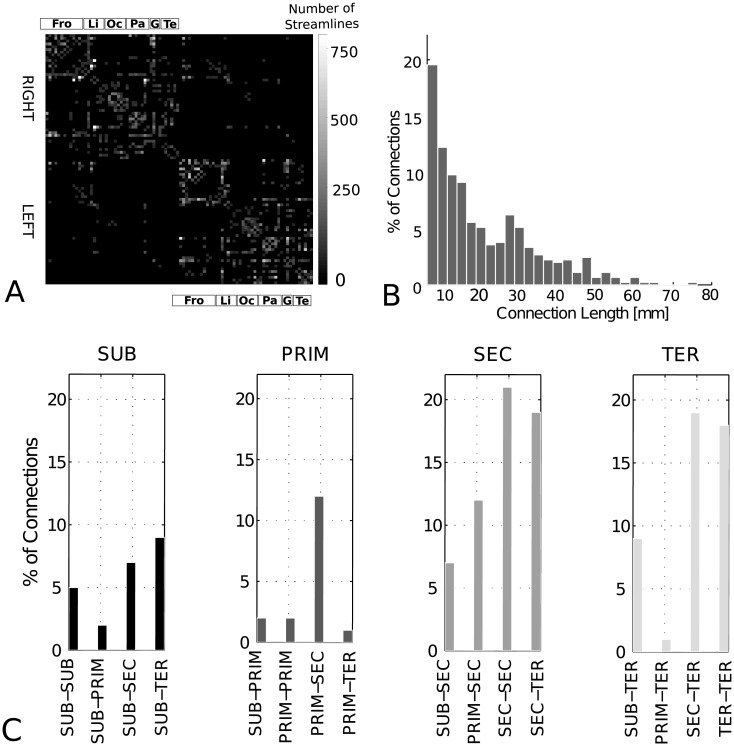
Connection properties. A: Weighted groupwise connectivity matrix including 450 connections present for > = 50% of the subjects. Color coding indicates the number of streamlines. ROIs are ordered per hemisphere (right: top, left: bottom) according to the atlas by Shi et al. [43]. Fro: frontal; Li: limbic; Oc: occipital; Pa: parietal; Te: temporal cortex and G: basal ganglia. B: Length distribution of the 450 connections. C: Connection repartition between the different groups. *SUB* in black, *PRIM* in dark gray, *SEC* in gray, *TER* in light gray.

Fig 1A represents the weighted group connectivity matrix for the 9 subjects, which has a density (percentage of non-zero entries) of 11.2% and no disconnected nodes. The colorbar indicates the number of streamlines for each connection. Fig 1B shows the connection length distribution for the connectivity network.

### Maturation biomarkers in GM and WM

In the first part of our study, we separate the 90 GM ROIs into 4 function-related groups: group *SUB* (n = 12, *n* number of ROIs) corresponds to subcortical nuclei, group *PRIM* (n = 12) represents the primary sensory and motor cortices, group *SEC* (n = 34) secondary processing areas and group *TER* (n = 32) higher order tertiary areas (S2 Table). Connection distribution among these groups is represented in Fig 1C. Subgroups SUB and SEC display connections to all other subgroups, while subgroup PRIM mainly connects to SEC and subgroup TER to all other groups but PRIM, which is anatomically meaningful.

**Fig 2:**
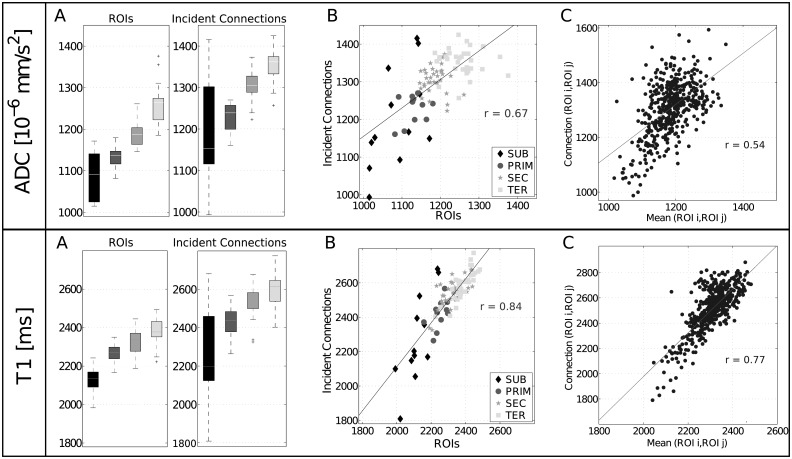
ROIs and incident connections. A: Distribution of ADC [10^−6^ mm/s^2^] (top) and T1 [ms] (bottom) values for each ROI group and the incident connections. B: Scatterplot for ADC (top) and T1 (bottom) in ROIs and their incident connections, for all ROIs. Pearson’s correlation: ADC *r* = 0.67, p < 10^−10^; T1 *r* = 0.84, p < 10^−10^. Groups: *SUB* (n = 12) in black (diamonds), *PRIM* (n = 12) in dark gray (circles), *SEC* (n = 34) in gray (stars), *TER* (n = 32) in light gray (squares). C: Correlation between mean ADC [10^−6^ mm/s^2^] (top) and T1 [ms] (bottom) of GM ROI pairs and average ADC value along the connecting WM tracts (ADC: Pearson *r* = 0.54, p < 10^−10^, T1: Pearson *r* = 0.77, p < 10^−10^; n = 450).

The distribution of mean ADC (top row) and T1 (bottom row) values in the 9 subjects is depicted for each group of ROIs in Fig 2A (left). We observe that ADC and T1 in the *SUB* group are lower than in the *PRIM* group, which in turn are lower than in the *SEC* and *TER* group and the latter have the highest values. A non parametric Jonckheere-Terpstra permutation (JT) analysis [57] confirms a significant ordered difference in these ADC (*JT* = 9.1, *p* ≈ 0.0) and T1 (*JT* = 7.5, p < 10^−10^) values. We also perform this analysis for the WM tracts incident to each ROI by computing the average over the incident connections (ADC: Fig 2A right, top, T1: Fig 2A right, bottom) and observe a comparable ordered increase in ADC (*JT* = 7.4, p < 10^−10^) and T1 (*JT* = 5.7, p < 10^−5^) across groups. Furthermore, significant correlation analyses demonstrate that ADC (*r* = 0.67, p < 10^−10^, Fig 2B top) and T1 (*r* = 0.84, p < 10^−10^, Fig 2B bottom) values in GM and WM are highly interdependent. Yet, this correlation was not present after randomization [58] of the connectivity matrix (ADC: *r* = -0.06, p = 0.56; T1: *r* = -0.006, p = 0.95).

A Pearson’s correlation analysis further shows that mean ADC of pairs of GM ROIs are correlated to the average values along the connecting WM tract (*r* = 0.53, p < 10^−10^, Fig 2C top). This is also the case for the T1 relaxation time (*r* = 0.77, p < 10^−10^, Fig 2C bottom). To account for possible proximity effects, analyses are reproduced on two subgroups of connections: *long* (> mean connection length, c = 179, c = number of connections) and *short* (< mean connection length, c = 271), yielding comparable results for ADC (*long*: *r* = 0.58, p < 10^−10^; *short*: *r* = 0.49, p < 10^−10^, S2 Fig) and T1 relaxation time (*long*: *r* = 0.79, p < 10^−10^; *short*: *r* = 0.75, p < 10^−10^, S3 Fig).

Taking a step further, we compare two groups of pairs of GM ROIs, those that are connected through a WM tract (450 pairs) and those that are not (3555 pairs). A Pearson’s correlation analysis outlines a significant correlation in ADC between GM ROI pairs if a structural link is present between them (*r* = 0.34, p < 10^−10^), while no association can be found if no connection is present (*r* = 0.03, *p* = 0.06). T1 relaxation time yields comparable results (connected: *r* = 0.25, p < 10^−5^; not connected: *r* = 0.009, *p* = 0.7). These results are reproduced at the single-subject level (S3 and S4 Tables).

### Modeling maturation

**Fig 3:**
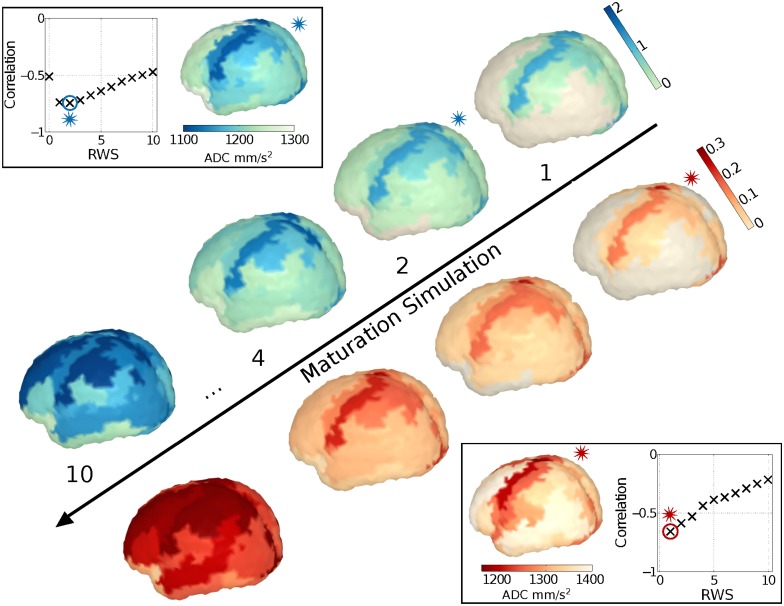
Modelled maturation scores projected on a standard brain surface for four representative simulated time points (RWSs): ROI in blue (top), connections in red (bottom). Box: Experimental data (ADC [10^−6^ mm/s^2^]) on brain surface and Pearson’s correlations with modelled maturation scores for RWS 1–10. The RWS with the best correlation is represented by a star.

In the second part of our study, we investigate possible mechanisms driving brain tissue maturation and the role of WM connectivity by means of a random walk model [56]. We consider the number of particle transits through each GM ROI or WM connection at each RWS as a score of local maturation. We observe that simulated maturation for ROIs (Fig 3: blue) and their incident connections (Fig 3: red) correlate negatively with the ADC values from our experimental data (Fig 3: box). Highest Spearman correlations between number of particle transits and ADC values are reached at early RWSs (ROIs: *r* = −0.75, p < 10^−10^ at RWS 2; incident connections: *r* = −0.68, p < 10^−10^ at RWS 1) and decrease progressively through the simulation. Correlation with T1 values shows a similar pattern (ROIs: r = −0.43, p < 10^−3^ at RWS 1; incident connections: r = −0.46, p < 10^−4^ at RWS 1).

As a validation, we perform the same analysis (i) using the same structural WM network, starting with a random sample of 10 GM regions as seeds and (ii) using a randomized structural network with preserved node degree distribution, starting from the same sensory areas. Both these randomization were repeated 50 times (50 random seedings and 50 network randomizations). In the first case, simulated maturation values showed no (ROIs, S4 Fig left) or weak (incident connections, S4 Fig right) correlation with the experimental data. In the second case, simulated maturation values showed weaker (ROIs, S5 Fig left) and no (incident connections, S5 Fig right) correlation with the experimental data.

**Fig 4:**
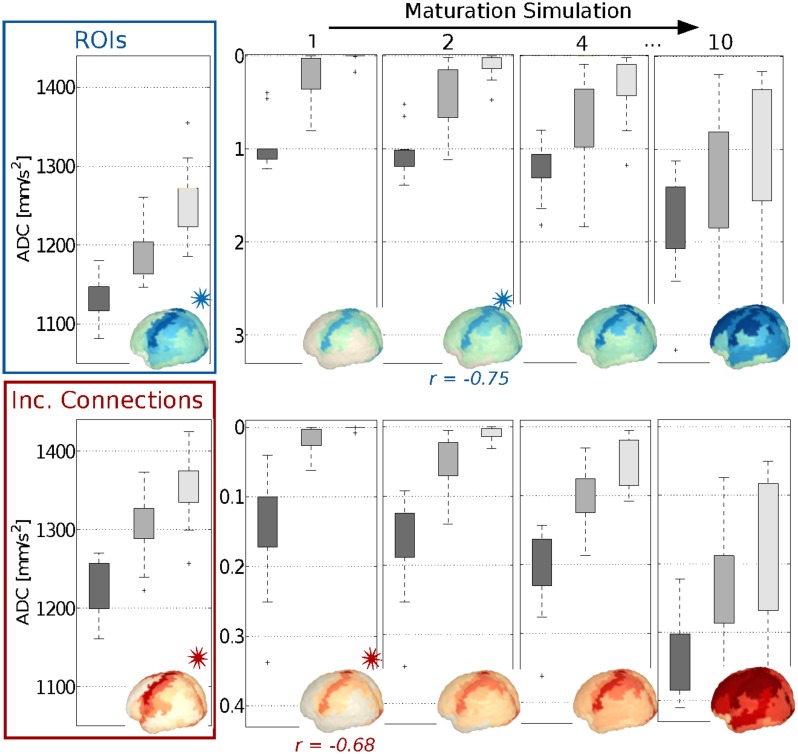
Histograms and brain surface plots for experimental ADC [10^−6^ mm/s^2^] values (left) and modelled maturation scores (right, four key RWSs) in ROI groups. Top: ROIs; bottom: incident connections. Highest Pearson’s correlations are indicated with a star for the corresponding RWS (and colour).

Simulated maturation values for ROIs and connections increase over progressive RWSs of the random walk, following an expected ordered pattern: areas from group *PRIM* have higher values than group *SEC* and further than group *TER*, which show the lowest values (Fig 4). This trend is confirmed by a JT analysis for each RWS (S5 Table). As expected, these differences lessen as the simulation reach later RWSs (decreasing JT statistics).

Note that subcortical areas were excluded from the correlation analysis, since *on one side* our random walk model exclusively explores the role of sensory input in network mediated maturation, and on the other side subcortical areas do not mature all at once [12,59,60] and their role in the maturation process is uncertain. This was also valid for the thalami, despite their known key role in the propagation and gating of sensory signals to primary sensory regions [61], because of their particular connection network. The various thalamic nuclei have heterogeneous connections to different cortical areas (including primary sensory, but also tertiary cortices), involved in information processing [62] and poor intra-thalamic connections. Hence, considering all thalamus nuclei as one ROI would imply creating an artificial hub. Although this is often done in adult structural and functional connectivity studies, it does not properly reflect functional anatomy, especially at early developmental stages, as all thalamo-cortical connections do no mature at the same time.

## Discussion

### Relation between GM and WM development

Our findings support a strong relationship between maturation of cortical areas and their underlying WM axonal circuits. The analysis of two MRI-based indicators of brain tissue maturation, namely ADC and T1 [36,39], reveals hierarchical increasing maturation levels from subcortical structures, to primary areas, to cortices involved in secondary and tertiary brain functions. A similar pattern is observed in the WM connections incident to these areas, suggesting a link between cortical maturation, long-range brain connectivity and WM myelination.

Our results are in agreement with the function-related maturation sequence previously described for GM structures in dissection studies where primary areas develop earlier than association and higher order processing areas [5,9,10]. Early studies examining WM myelination reported comparable findings [12,13]. However later research describes myelination as following a spatial rather than a functional and connectional gradient [14,15,39].

Early connectivity studies investigate the co-variation between cortical morphology measures (e.g., cortical thickness, surface area) in different brain regions and report highly organized correlation patterns, coinciding with the presence of underlying gross anatomical tracts [63–65]. These findings were reproduced in a developmental approach by Raznahan and colleagues who showed that cortical thickness measures for different brain regions belonging to the default mode network and one task oriented network can be predicted from well-described patterns of cortical functional and WM interconnectivity [66]. The existence of a strong relationship between GM and WM maturation is also supported by evolutionary studies. For example, Barton and Harvey highlight that the coordination of brain anatomical changes follows known patterns of structural connectivity [67].

Despite these encouraging results, explicit reports on the relationship between maturation parameters in GM and WM remain few and their results inconsistent. Cortical ADC is related to ADC in the underlying WM regions in one primary, two secondary and one tertiary brain region [68]. But a whole-brain investigation of cortical thickness and myelin content did not highlight such a relationship [69].

Compared to these findings, the strength of our study lies in the joint assessment of early stages of GM and WM maturation over the whole cerebrum at the level of (i) single structures and (ii) subsystems consisting of pairs of GM regions and their anatomical link. We show that ADC and T1 values in GM areas are consistently related to values along the axonal fiber bundles connecting them. Others, using a T2-based MRI sequence and DTI, noted a comparable relation for functional subsystems in temporal [70] and pre-frontal [71] regions. Additionally, a recent study showed a correlation between decrease in cortical thickness (interpreted as maturation) in frontal cortical regions and increase in fractional anisotropy in the WM tracts initiated from these regions, in school-age children [72]. These results point out a strong interdependence between the development of GM structures and the underlying WM connectivity network. Correlation analyses indicate that GM areas are not developing independently. Indeed we observe that ADC and T1 in GM regions connected through an anatomical link are highly related. On the same line, correlations of T2-measures [70] and cortical thickness [63,66] were reported in connected cortical regions involved in language processing and in two functional networks. Furthermore, shared expression signatures for genes involved in neuronal development and axon guidance also seem to depend on structural connectivity in different regions of the rat brain [73]. Altogether, this points to an important role of structural WM connections during the cortical maturation process.

### A connectivity-based model for brain maturation

A large body of evidence suggests that sensory-driven neural activity plays a key role in shaping the maturation of GM and WM cerebral structures at the cellular level ([74], for a review). Neural signals, be they initially endogenous and later on exogenous, are relayed hierarchically through thalamo-cortical pathways from primary receiving areas to higher order processing regions [75,76]. Together with our findings on the interplay between GM and WM maturation, this suggests that the connectivity network conveys signals implicated in maturation processes.

This work proposes a simple random-walk model to explore the role of the WM network on the aspects of the maturation process that are mechanistically related to sensory inputs. In our model, maturation particles (i.e., random walkers) are initiated in cortical sensory areas and left to diffuse across the WM network. A similar computational approach was recently used to mimic viral-like spreading of neurodegenerative diseases across the structural WM network [77,78]. Indeed we observe that simulated GM and WM maturation scores reproduce experimental early maturation patterns, and they increase hierarchically in time following function-related developmental patterns reported in literature [5,9,10].

The idea that tissues' maturation could progress in a hierarchical fashion from lower-order sensory-receiving areas to the yet immature higher-order areas in their vicinity was already proposed by Guillery [79]. Our findings suggest that the WM connectivity backbone could play a key role in relaying maturation signals. It is realistic to think that during early development, the callow brain network mainly conveys coherent sensory information to higher processing areas, which would promote the maturation of related axonal pathways and connected GM regions. In line with this, connections between lower level unimodal regions strengthen before connections towards association areas, and further on to higher order processing regions [80]. Altogether, the progression of maturation from lower order areas to higher processing regions simulated with our model resembles the evolution of GM density measures between 5 and 20 years reported by Gogtay [81]. Finally, numerous studies on prematurity support the concept that WM connectivity is critical for the development of connected cortices and its lesion leads to histological disorganization of connected GM areas [82–84].

### Technical limitations

The main limits of this study are related to the characteristics of the investigated population, particularly the small sample size and the prematurity condition. On one side, premature birth is associated with delayed and altered microstructural development of cortical GM [85] and WM circuitry ([86], for a review). On the other side, these changes vary with gestational age and diffuse aggressions (e.g., nutrition, infection), which are common in this population [87]. We argue that the effects pursued in this study are coarse macroscopic maturation changes outweighing subtle inter-population differences and should also be observable in prematurely born children with ‘cerebral low-risk’, scanned at term equivalent age as in normal term born kids. Consistently, subject-level analyses reproduce group-level findings, excluding the presence of outliers in our cohort.

Imaging neonates is both technically and ethically challenging. First, scanning normal term newborns is ethically debatable, reason why we focused on prematurely born babies who deserve through their condition an MRI examination. Second, scans had to be done without sedation, increasing the chance of motion artefacts. Additionally, tissue contrasts in neonatal brain differ notably from those of adult brains [88]. This difference can have an impact on image processing steps. In this work, we performed careful quality checks and post-processing motion correction of our data. However, we did not quantify mean relative frame-wise displacement between interspersed non-weighted diffusion volumes as has been done by others [89], but outlier rejection was performed by a senior pediatric neuroradiologist who checked all sequences visually during acquisition and had the ones with movement artifacts repeated. Six subjects were excluded because of poor data quality due to excessive motion during scans.

Another limitation of this study concerns the estimation of tissue maturation levels using MRI. Variations in MRI signals result from multiple and inter-dependent cellular processes [90], making the mapping between MRI parameters and cellular maturation processes challenging. ADC and T1 measures relate to multiple neurobiological aspects of the maturation process. ADC measures intra- and extra-cellular water mobility, which is influenced by dendritic arborization, axonal packing and myelin sheath thickness, while T1 provides information about lipid and macromolecules concentration associated with myelination [36,39,60,91,92]. Accordingly, a combination of these MRI parameters is well suited for the study of brain maturation in vivo [93]. Furthermore, partial volume effects and cortical ingrowing axons may contribute to the cortical ADC and T1 values in areas located at the interface between cortical GM and subcortical WM. We limited these effects by excluding GM voxels with ADC and T1 values differing by more than one standard deviation from the ROI mean from the analysis. For a more accurate estimation of the tissues maturation level, advanced MRI sequences tailored to WM microstructural investigation [94] are needed. However, these methodological approaches are still in their early stage and the applicability to newborns remains difficult due to the long acquisition time.

In order to limit the scanning time, a DTI sequence was chosen for this study. It is well known that the combination of DTI and deterministic tractography could under-estimate the degree of inter-regional connectivity between ROIs linked by complex and long WM trajectories [95,96]. However, this rather conservative approach limits the number of retrieved false positive connections [96] and seems well-suited for the correlation and random-walk analyses performed in the present study [97]. Indeed, this study concentrates on uncovering coarse macroscopic maturation mechanisms and does not attempt to provide a precise description of these.

Finally, we compared our random walk model with experimental MRI biomarkers from a single age only. The relatively coarse developmental steps of the model, along with the early developmental stage of the studied population could explain higher correlations at early RWSs. Still, although qualitative observations demonstrate a clear link with developmental patterns described in the literature, extension of this study to control populations across lifespan could yield further biological insights into neurodevelopmental mechanisms.

## Supporting information

S1 Fig Anatomical ROI masks for all 9 subjects. The same transverse (first column), sagittal (second column) and coronal (third column) are displayed. Color-coding reflects single ROIs and corresponding ROIs for each hemisphere are displayed in the same colour.
(EPS)

S2 Fig Correlation between mean ADC [10^−6^ mm/s^2^] of GM ROI pairs and average ADC value along the connecting WM tracts (Fig 2C top) for *short* connections (< mean connection length, c = 271, c = number of connections): Pearson *r* = 0.49, p < 10^−10^; and long connections (> mean connection length, c = 179): Pearson *r* = 0.58, p < 10^−10^. (EPS)

S3 Fig Correlation between mean T1 [ms] of GM ROI pairs and average ADC value along the connecting WM tracts (Fig 2C bottom) for *short* connections (< mean connection length, c = 271, c = number of connections): Pearson *r* = 0.75, p < 10^−10^; and long connections (> mean connection length, c = 179): Pearson *r* = 0.79, p < 10^−10^. (EPS)

S4 Fig Maturation simulation null model (1): Correlation between experimental and simulated data for 50 random seedings (random sampling of 10 regions from all possible ROIs): ROIs (left), incident connections (right). (EPS)

S5 Fig Maturation simulation null model (2): Correlation between experimental and simulated data for 50 randomizations of the structural network with preserved node degree distribution: ROIs (left), incident connections (right). Note that the simulations starts from the same sensory areas as the original maturation simulation.
(EPS)

S1 Table Subjects’ characteristics: sbj indicates subject number; GA, gestational age; BW: birthweight; M, months; IVH, intraventricular hemorrhage grading according to Papile [98] on cerebral ultrasound (3 infants presented with IVH: 2 with grade I and 1 with bilateral grade II). Not shown here, only one infant (Sbj 3) presented with a small unilateral cerebellar hemorrage. PVL indicates periventricular leucomalacia grading according to L. de Vries [99] on cerebral ultrasound. MDI and PDI are mental and psychomotor developmental indices from Bayley Scales of Infant Development II edition, norm mean (SD) is 100 (15). The values obtained are consistent with expected findings of good evolving preterm babies. All these babies had a normal neurological exam at term equivalent age (TEA). According to the Kidokoro score [100,101] only 2 infants had a mildly abnormal score (5 and 7) wheareas all others presented with a normal score (1–3). Values are presented in mean ± SD, except for the Kidokoro score* (median and range).
(DOCX)

S2 Table ROI allocation for each group (for one hemisphere). Note that the ROI distribution is symmetric between both hemispheres.
(DOCX)

S3 Table ADC [10^−6^ mm/s^2^] single subject’s values. (DOCX)

S4 Table T1 [ms] single subject’s values. (DOCX)

S5 Table Non parametric Jonckheere-Terpstra permutation analysis (JT) for four key random walk steps (RWSs). (DOCX)
