# Supplementary material for: Exploring the role of white matter connectivity in cortex maturation
Source: PLoS One. 2017 May 17;12(5):e0177466. doi: 10.1371/journal.pone.0177466 (PMC5435226; doi:10.1371/journal.pone.0177466)
Supplement: S1 Table — Not shown here, only one infant (Sbj 3) presented with a small unilateral cerebellar hemorrage. PVL indicates periventricular leucomalacia grading according to L. de Vries [99] on cerebral ultrasound. MDI and PDI are mental and psychomotor developmental indices from Bayley Scales of Infant Development II edition, norm mean (SD) is 100 (15). The values obtained are consistent with expected findings of good evolving preterm babies. All these babies had a normal neurological exam at term equivalent age (TEA). According to the Kidokoro score [100,101] only 2 infants had a mildly abnormal score (5 and 7) wheareas all others presented with a normal score (1–3). Values are presented in mean ± SD, except for the Kidokoro score* (median and range). (DOCX) [file pone.0177466.s006.docx]

**S1 Table: Subjects’ characteristics**

| **Sbj** | **GA** | **GA at scan** | **IVH**  **grade** | **PVL**  **grade** | **BW**  **(in grams)** | **Kidokoro score MRI** | **MDI 18M** | **PDI 18M** |
| --- | --- | --- | --- | --- | --- | --- | --- | --- |
| 1 | 28 ^2^/_7_ | 39 5/7 | 1 | 1 | \| 860 \| \| --- \| | 3 | 85 | 83 |
| 2 | 27 ^4^/_7_ | 41 5/7 | 0 | 1 | \| 1180 \| \| --- \| | 5 | 95 | 95 |
| 3 | 26 ^4^/_7_ | 43 2/7 | 2 | 0 | \| 830 \| \| --- \| | 3 | 98 | 84 |
| 4 | 29 ^2^/_7_ | 42 5/7 | 0 | 0 | \| 1040 \| \| --- \| | 7 | 71 | 79 |
| 5 | 29 ^0^/_7_ | 42 3/7 | 0 | 0 | \| 1230 \| \| --- \| | 1 | 105 | 95 |
| 6 | 27 ^3^/_7_ | 41 1/7 | 0 | 0 | \| 947 \| \| --- \| | 3 | 79 | 75 |
| 7 | 28 ^5^/_7_ | 38 5/7 | 0 | 0 | \| 1180 \| \| --- \| | 3 | 89 | 82 |
| 8 | 28 ^1^/_7_ | 40 4/7 | 1 | 0 | \| 759 \| \| --- \| | 1 | 85 | 74 |
| 9 | 26 ^0^/_7_ | 41 1/7 | 0 | 1 | \| 673 \| \| --- \| | 1 | 79 | 83 |
| *mean* | 27.1  ±1/7 | 41  ±2/7 |  |  | \| 966,56 \| \| --- \| \| ±201,73 \| | * 3 (1-7) | 87.3 ±10.6 | 83.3  ±7.5 |
