# Supplementary material for: Exploring the role of white matter connectivity in cortex maturation
Source: PLoS One. 2017 May 17;12(5):e0177466. doi: 10.1371/journal.pone.0177466 (PMC5435226; doi:10.1371/journal.pone.0177466)
Supplement: S2 Table — Note that the ROI distribution is symmetric between both hemispheres. (DOCX) [file pone.0177466.s007.docx]

**S2 Table: ROI allocation for each group (for one hemisphere)**:

| SUB (n= 6) | PRIM (n=6) | SEC (n = 17) | TER (n = 16) |
| --- | --- | --- | --- |
| Hippocampus | Precentral Gyrus | Inferior frontal gyrus (opercular) | Superior frontal gyrus |
| Amygdala | Rolandic operculum | Supplementary motor area | Orbitofrontal cortex (superior) |
| Caudate | Calcarine cortex | Insula | Middle frontal gyrus |
| Putamen | Postcentral gyrus | Middle cingulate gyrus | Orbitofrontal cortex (middle) |
| Pallidum | Paracentral lobule | Posterior cingulate gyrus | Inferior frontal gyrus (triangular) |
| Thalamus | Heschl gyrus | Parahippocampal | Orbitofrontal cortex (inferior) |
|  |  | Cuneus | Olfactory |
|  |  | Lingual gyrus | Superior frontal gyrus (medial) |
|  |  | Superior occipital gyrus | Orbitofrontal cortex (medial) |
|  |  | Middle occipital gyrus | Rectus gyrus |
|  |  | Inferior occipital gyrus | Anterior cingulate gyrus |
|  |  | Fusiform gyrus | Angular gyrus |
|  |  | Superior parietal gyrus | Temporal pole (superior) |
|  |  | Inferior parietal lobule | Middle temporal gyrus |
|  |  | Supramarginal gyrus | Temporal pole (middle) |
|  |  | Precuneus | Inferior temporal gyrus |
|  |  | Superior temporal gyrus |  |

Note that the ROI distribution is symmetric between both hemispheres.
