# Supplementary material for: Exploring the role of white matter connectivity in cortex maturation
Source: PLoS One. 2017 May 17;12(5):e0177466. doi: 10.1371/journal.pone.0177466 (PMC5435226; doi:10.1371/journal.pone.0177466)
Supplement: S3 Table — (DOCX) [file pone.0177466.s008.docx]

**S3 Table A and B: ADC [10^-6^ mm/s^2^] single subject’s values**

A.

| **Sbj** | **ROI**  (fig.2A top left) | | **Incident connections**  (fig.2A top right) | | **ROI vs. inc. connections**  (fig.2B top) | | **Mean ROI vs connecting fibre**  (fig.2C top) | |
| --- | --- | --- | --- | --- | --- | --- | --- | --- |
|  | *JT* | *p* | *JT* | *p* | *r* | *p* | *r* | *p* |
| 9 | 8.56 | <10^-10^ | 6.28 | <10^-5^ | 0.63 | <10^-10^ | 0.39 | <10^-10^ |
| 8 | 8.63 | <10^-10^ | 7.28 | <10^-10^ | 0.59 | <10^-5^ | 0.47 | <10^-10^ |
| 7 | 7.96 | <10^-10^ | 6.03 | <10^-5^ | 0.57 | <10^-5^ | 0.53 | <10^-10^ |
| 6 | 6.30 | <10^-5^ | 5.21 | <10^-5^ | 0.54 | <10^-5^ | 0.47 | <10^-10^ |
| 5 | 7.30 | <10^-10^ | 6.08 | <10^-5^ | 0.61 | <10^-5^ | 0.33 | <10^-5^ |
| 4 | 8.39 | <10^-10^ | 4.46 | <10^-5^ | 0.53 | <10^-5^ | 0.50 | <10^-10^ |
| 3 | 7.59 | <10^-10^ | 7.05 | <10^-10^ | 0.63 | <10^-10^ | 0.40 | <10^-10^ |
| 2 | 8.33 | <10^-10^ | 7.26 | <10^-10^ | 0.74 | <10^-10^ | 0.48 | <10^-10^ |
| 1 | 7.47 | <10^-10^ | 6.28 | <10^-5^ | 0.61 | <10^-5^ | 0.55 | <10^-10^ |

B.

| **Sbj** | **connected** | | **unconnected** | |
| --- | --- | --- | --- | --- |
|  | *r* | *p* | *r* | *p* |
| 9 | 0.29 | <10^-5^ | -0.01 | 0.47 |
| 8 | 0.37 | <10^-10^ | -0.01 | 0.47 |
| 7 | 0.26 | <10^-5^ | 0.02 | 0.25 |
| 6 | 0.35 | <10^-10^ | -0.02 | 0.31 |
| 5 | 0.40 | <10^-10^ | -0.02 | 0.22 |
| 4 | 0.23 | <10^-5^ | 0.005 | 0.76 |
| 3 | 0.38 | <10^-10^ | 0.04 | 0.02 |
| 2 | 0.34 | <10^-10^ | 0.01 | 0.49 |
| 1 | 0.36 | <10^-10^ | -0.02 | 0.34 |
