# Supplementary material for: Exploring the role of white matter connectivity in cortex maturation
Source: PLoS One. 2017 May 17;12(5):e0177466. doi: 10.1371/journal.pone.0177466 (PMC5435226; doi:10.1371/journal.pone.0177466)
Supplement: S4 Table — (DOCX) [file pone.0177466.s009.docx]

**S7 Table A and B: T1 [ms] single subject’s values**

A.

| **Sbj** | **ROI**  (fig.S2A bottom left) | | **Incident connections**  (fig.S2A bottom right) | | **ROI vs. inc. connections**  (fig.S2B bottom) | | **Mean ROI vs connecting fibre**  (fig.S2C bottom) | |
| --- | --- | --- | --- | --- | --- | --- | --- | --- |
|  | *JT* | *p* | *JT* | *p* | *r* | *p* | *r* | *p* |
| 9 | 7.11 | <10^-10^ | 4.20 | <10^-4^ | 0.60 | <10^-5^ | 0.65 | <10^-10^ |
| 8 | 6.88 | <10^-10^ | 4.78 | <10^-5^ | 0.71 | <10^-10^ | 0.70 | <10^-10^ |
| 7 | 4.11 | <10^-4^ | 3.15 | <10^-3^ | 0.62 | <10^-10^ | 0.65 | <10^-10^ |
| 6 | 7.60 | <10^-10^ | 6.00 | <10^-5^ | 0.77 | <10^-10^ | 0.65 | <10^-10^ |
| 5 | 7.01 | <10^-10^ | 4.24 | <10^-4^ | 0.63 | <10^-10^ | 0.65 | <10^-10^ |
| 4 | 8.32 | <10^-10^ | 3.16 | <10^-3^ | 0.59 | <10^-5^ | 0.62 | <10^-10^ |
| 3 | 8.10 | <10^-10^ | 4.74 | <10^-5^ | 0.60 | <10^-5^ | 0.66 | <10^-10^ |
| 2 | 7.18 | <10^-10^ | 5.79 | <10^-5^ | 0.75 | <10^-10^ | 0.64 | <10^-10^ |
| 1 | 7.41 | <10^-10^ | 6708 | <10^-10^ | 0.74 | <10^-10^ | 0.72 | <10^-10^ |

B.

| **Sbj** | **connected** | | **unconnected** | |
| --- | --- | --- | --- | --- |
|  | *r* | *p* | *r* | *p* |
| 9 | 0.23 | <10^-5^ | -0.03 | 0.07 |
| 8 | 0.32 | <10^-10^ | -0.03 | 0.05 |
| 7 | 0.30 | <10^-5^ | -0.04 | 0.02 |
| 6 | 0.43 | <10^-10^ | -0.01 | 0.54 |
| 5 | 0.30 | <10^-5^ | -0.02 | 0.34 |
| 4 | 0.18 | <10^-3^ | -0.003 | 0.88 |
| 3 | 0.32 | <10^-5^ | -0.005 | 0.74 |
| 2 | 0.26 | <10^-5^ | 0.003 | 0.86 |
| 1 | 0.31 | <10^-5^ | -0.03 | 0.07 |
