# Supplementary material for: Exploring the role of white matter connectivity in cortex maturation
Source: PLoS One. 2017 May 17;12(5):e0177466. doi: 10.1371/journal.pone.0177466 (PMC5435226; doi:10.1371/journal.pone.0177466)
Supplement: S5 Table — (DOCX) [file pone.0177466.s010.docx]

**S10 Table:** Non parametric Jonckheere-Terpstra permutation analysis (JT) for four key random walk steps (RWSs).

| RWS | ROIs | | Connections | |
| --- | --- | --- | --- | --- |
|  | *JT* | *p-value* | *JT* | *p-value* |
| 1 | 7.5 | <10^-10^ | 7.6 | <10^-10^ |
| 2 | 7.3 | <10^-10^ | 7.9 | <10^-10^ |
| 4 | 5.9 | <10^-5^ | 6.7 | <10^-5^ |
| 10 | 3.5 | <10^-2^ | 4.6 | <10^-4^ |
